# Outcomes of dual‐chamber implantable cardioverter defibrillator for left bundle branch area pacing: A systematic review of literature

**DOI:** 10.1111/anec.13098

**Published:** 2023-11-23

**Authors:** Muhammad Ahmad, Saffa Nadeem, Hafiz Ahmed Raza, Abdul Wasey Hashmi, Fawad Talat, Deepak Kumar, Syed Muhammad Jawad Zaidi, Amin Mehmoodi, Jahanzeb Malik

**Affiliations:** ^1^ Department of Medicine Al Saeed Medical Complex Rahim Yar Khan Pakistan; ^2^ Department of Cardiology Multan Institute of Cardiology Multan Pakistan; ^3^ Department of Emergency Medicine Social Security Hospital Sheikuphura Pakistan; ^4^ Department of Medicine Shalamar Medical and Dental College Lahore Pakistan; ^5^ Department of Medicine King Edward Medical University Lahore Pakistan; ^6^ Department of Medicine Jinnah Sindh Medical University Karachi Pakistan; ^7^ Department of Medicine Ibn e Seena Hospital Kabul Afghanistan; ^8^ Department of Cardiovascular Research Cardiovascular Analytics Group Islamabad Pakistan

**Keywords:** cardiac anatomy, clinical, implantable devices – ventricular tachycardia/fibrillation

## Abstract

**Objective:**

This systematic review of literature aimed to evaluate the safety and efficacy of dual‐chamber ICDs for LBBAP in patients with left bundle branch block (LBBB).

**Methods:**

Digital databases were searched systematically to identify studies reporting the left bundle branch area pacing (LBBAP) with implantable cardioverter defibrillator (ICD) placement in patients with LBBB. Detailed study and patient‐level baseline characteristics including the type of study, sample size, follow‐up, number of cases, age, gender, and baseline characteristics were abstracted.

**Results:**

In a total of three studies, 34 patients were included in this review. There was a significant improvement reported in QRS duration in all studies. The mean QRS duration at baseline was 170 ± 17.4 ms, whereas the follow‐up QRS duration at follow‐up was 121 ± 17.3 ms. Two studies reported a significant improvement of 50% in LVEF from baseline. No lead‐related complications or arrhythmic events were recorded in any study. The findings of the systematic review suggest that dual‐chamber ICD for LBBAP is a promising intervention for patients with heart conditions.

**Conclusion:**

The procedure offers significant improvements in QRS duration and LVEF, and there were no lead‐related complications or arrhythmic events recorded in any of the studies.

## Graphical abstract

Ports of LBBAP‐ICD.

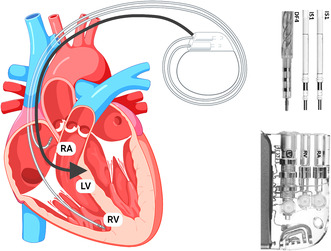

## 1 INTRODUCTION

Implantable cardioverter defibrillators (ICDs) have been an important therapy for patients with heart failure, ventricular arrhythmias, and other cardiac conditions (Borne et al., 2013). Recent advances in technology have led to the development of dual‐chamber ICDs, which offer the ability to pace both the right and left ventricles of the heart (Linde et al., 2012). Left bundle branch area pacing (LBBAP) is a relatively new technique that has shown promising results in patients with left bundle branch block (LBBB) (Jastrzębski et al., 2022). The use of dual‐chamber ICDs for LBBAP is an emerging treatment option that has been the subject of some studies in recent years (Clementy et al., 2022; Huybrechts et al., 2023; Ponnusamy et al., 2022). More recently, multisite pacing with a combination of normal epicardial LV pacing, via the coronary sinus, and LBBAP has been proposed to further improve cardiac resynchronization therapy (CRT) (Clementy et al., 2022).

This systematic review of literature aimed to evaluate the safety and efficacy of dual‐chamber ICDs for LBBAP in patients with LBBB. The findings of this systematic review will provide important insights into the use of dual‐chamber ICDs for LBBAP in patients with LBBB. It will inform clinicians and researchers about the safety and efficacy of this emerging treatment option and guide the development of future clinical trials in this area.

## 2 METHODS

This review was followed according to the Preferred Reporting Items for Systematic Reviews and Meta‐Analyses (PRISMA) statement (Liberati et al., 2009). Data retrieved from the included articles are available in the references section of Table 1.

**TABLE 1:** Study characteristics.

Author name/year	Country	Study type	Sample size	Males (%)	Mean age	Pacing parameters	Baseline QRS duration	Follow‐up QRS duration	Baseline LVEF	Follow‐up LVEF	Arrhythmic event recorded	Key outcomes	Study quality
Huybrechts et al. (2023)	Belgium	Nonrandomized prospective cohort study	5	4 (80%)	57 ± 16.5 years	Acute LBBA pacing threshold and R‐wave amplitudes were 0.80 ± 0.60 V at 0.4 milliseconds and 7.0 ± 2.7 mV	170.0 ± 7.3 ms	121.3 ± 8.3 ms	28.8% ± 11.7%	Not reported	No LBBA lead‐related complications	ICD with LBB pacing showed significant improvement in electrocardiographic parameters.	*****
Ponnusamy et al. (2022)	India	Prospective cohort (single centered	11	72%	58.2 ± 12.5 years	Pacing threshold was 0.58 ± 0.36 V at 0.5 ms pulse width, sensed R‐wave amplitude was 9.9 ± 5.8 mV	176.1 ± 21.3 ms	118.4 ± 18.7 ms	28.9 ± 3.7%	47.4 ± 10.4%	One patient had atrial and one had ventricular tachycardia which were optimized at follow‐up.	The combinate ICD with LBB pacing can obviate the need for a CRT‐D pulse Generator.	****
Clementy et al. (2022)	France	Prospective cohort	10	60%	68 ± 3	Pacing threshold was 0.75 and 0.50 V at a 0.4 ms pulse width	164 (24)	124 (25)	29 (8)	44 (12)	No complication reported	Acute ventricular arrhythmia sensing and defibrillation can be performed via a single LBBAP lead connected to a dual‐chamber ICD	******

Asterix means one point as per newcastle ottawa scale for quality assessment, more asterix means more qualified study.

### 2.1 Search strategy and selection criteria

PubMed, Embase, CINAHL, Web of Science, and Cochrane databases were searched using the medical subject headings (MeSH) keywords to find out the studies of interest. There were no time filters or language restrictions in place and relevant articles were also screened using the backward snowballing technique. The following MeSH terms were used: “left bundle branch area pacing” AND “implantable cardioverter defibrillator” AND “cardiac resynchronization therapy” AND ‘study outcomes” AND “left ventricular ejection fraction”. All subsets were combined systematically by Boolean operators into the Covidence library.

Two investigators (J.M. and S.N.) independently reviewed the abstracts and titles of the original articles extracted as an initial search and selected articles that reported the outcomes of ICD in LBBAP, including randomized controlled trials, nonrandomized trials, and observational studies. All data were validated by the first author and in case of missing data, authors of the original article were contacted. The last search ended on May 1, 2023.

### 2.2 Data extraction and analysis

The above‐mentioned authors extracted the data about LBBAP and ICD as the study and detailed patient‐level baseline characteristics, including the type of study, sample size, baseline and follow‐up QRS, arrhythmic events recorded, and key outcomes were abstracted.

The statistical analysis was performed using the Statistical Package for Social Sciences (SPSS) version 26 (IBM Corp., Armonk, NY, USA.) Continuous data were presented as mean and standard deviation (SD), while categorical data were expressed with frequency (n) and percentages (%).

### 2.3 Quality assessment

The overall study quality was not an exclusion criterion as the articles were less on our subject question and the Newcastle‐Ottawa Scale was used for assessing the studies included in this review. The quality of the included studies is presented in Table 1.

## 3 RESULTS

### 3.1 Search results

**FIGURE 1:**
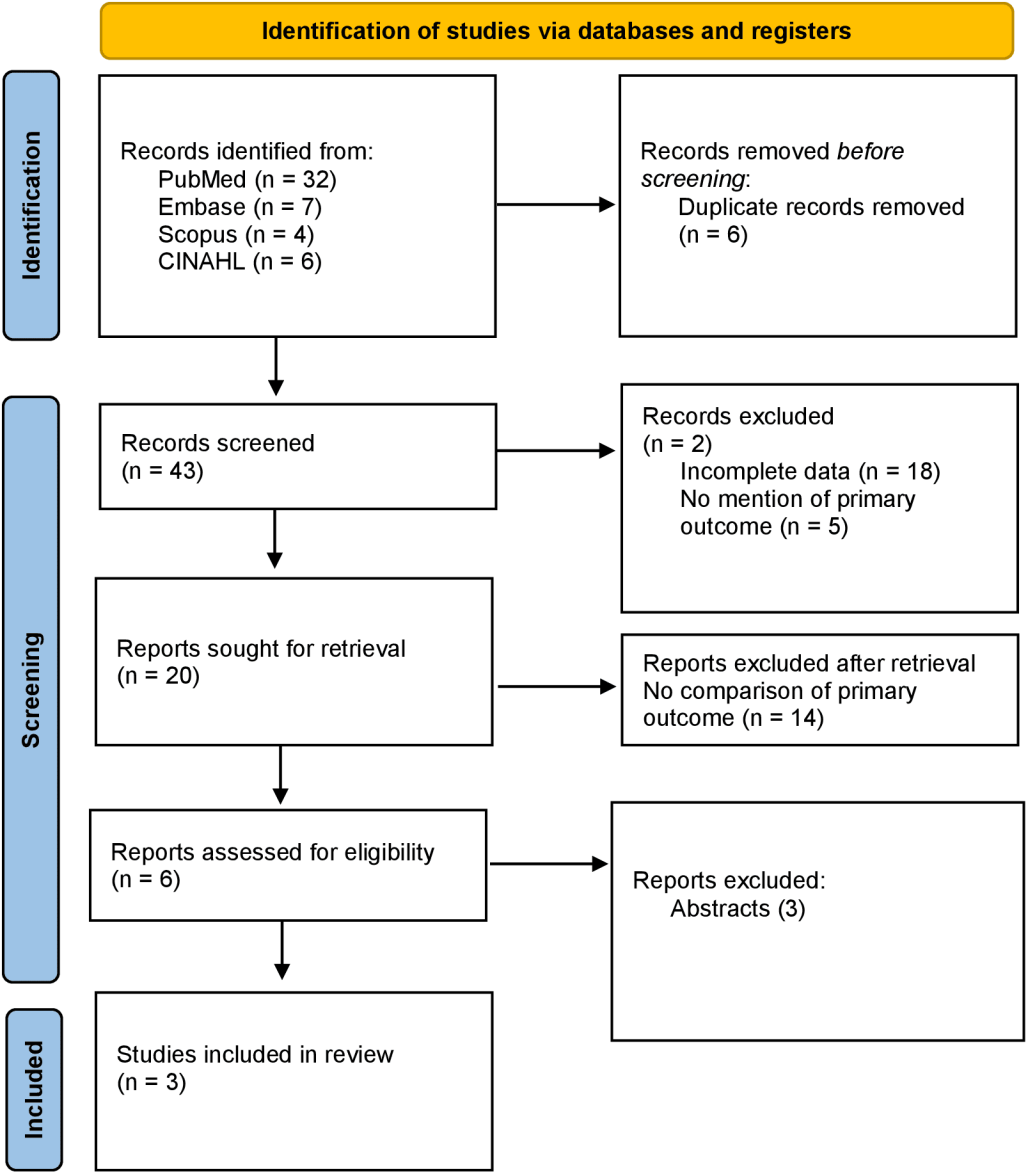
PRISMA flowchart.

With an initial search result, a total of 43 articles of interest were retrieved. After the removal of duplication and irrelevant articles (23), a total of 20 articles were assessed for eligibility. Finally, a total of three studies were deemed eligible for systematic review. All included studies were prospective cohort studies. The three studies were from India, Belgium, and France. The PRISMA flow chart is shown in Figure 1.

### 3.2 Study characteristics and outcomes

A total of 34 patients underwent ICD implantation for Left bundle branch pacing of which 18 (69.2%) were males. The mean age of all the study participants was 61.1 ± 9.1 years. As far as pacing parameters were concerned, the mean pacing threshold was 0.71 V and pulse width ranged between 0.4 and 0.5 mV. There was a significant improvement reported in QRS duration in all studies. The mean QRS duration at baseline was 170 ± 17.4 ms, whereas the follow‐up QRS duration at follow‐up was 121 ± 17.3 ms. Two studies reported a significant improvement of 50% in LVEF from baseline. No lead‐related complications or arrhythmic events were recorded in any study. These details of study‐wise parameters are delineated in Table 1. Huybrechts et al. (2023) showed that ICD with LBBAP significantly improves electrocardiographic parameters. Ponnusamy et al. (2022) demonstrated that the combination of ICD with LBBAP can obviate the need for CRT‐D Pulse Generator. Clementy et al. (2022) showed that acute ventricular arrhythmia sensing and defibrillation can be performed via a single LBBAP lead connected to a dual‐chamber ICD.

## 4 DISCUSSION

### 4.1 Main findings

The main findings of this systematic review are as follows: (1) ICD by LBBAP was successful in more than 60% of CRT‐D‐eligible patients, (2) LBBAP provided stable device parameters for arrhythmia monitoring, and (3) LBBAP with ICD maybe a cost‐effective resynchronization therapy at reduced fluoroscopy time duration and radiation dose (Clementy et al., 2022; Huybrechts et al., 2023; Ponnusamy et al., 2022). A systematic review has been done for the first time to consolidate this limited data on the use of ICDs for CRT patients with left systolic dysfunction and a complete LBBB. At the same time, VF sensing appears to be similar in the right ventricular chamber in the deep interventricular septum. There was a significant improvement reported in QRS duration and LVEF. LBBAP has been suggested as an alternative as effective as CRT in patients with LBBB and non‐LBBB morphology. In patients with no bi‐ventricular pacing, ICD with LBBAP provides superior electrical resynchronization, The success rate is variable in the three studies included in this review due to the highly selected population with ICD indication with LBBAP. The review included three prospective cohort studies from India, Belgium, and France, involving a total of 26 patients who underwent ICD implantation for LBBAP. The study participants had a mean age of 61.1 ± 9.1 years, and 69.2% were males. The review found that there was a significant improvement in QRS duration in all the studies. The mean QRS duration at baseline was 170 ± 17.4 ms, while the follow‐up QRS duration was 121 ± 17.3 ms. Additionally, two studies reported a significant improvement of 50% in left ventricular ejection fraction (LVEF) from baseline (Clementy et al., 2022; Ponnusamy et al., 2022). The mean pacing threshold was 0.71 V, and the pulse width ranged between 0.4 and 0.5 mV. No lead‐related complications or arrhythmic events were recorded in any of the studies.

### 4.2 Conduction system anatomy

Tawara (1906) gave the first description of the atrioventricular (AV) node, the presence of His bundle, and its right and left branches (Knorre, 2006). His bundle has two segments—the penetrating portion and the branching portion (Massing & James, 1976). The branching portion reaches 5–10 mm from posterior fibers to the point, where LB completely branches out and the right bundle begins at his point (Elizari, 2017). Hence, this is commonly termed pseudo‐bifurcation. The proximal portion of LB continues beneath the LV endocardium to form a wider target for pacing compared with a narrow His bundle. There it divides into anterior and posterior fascicles, each heading toward their respective papillary muscle heads. These fascicles give rise to Purkinje fibers, which arborize into the ventricular myocardium (Duan et al., 2017).

### 4.3 Left bundle branch area pacing

LBBAP means directly capturing the left main bundle or one of its fascicles along with the LV septal myocardium at the lowest output (<1 V at 0.5‐ms pulse width) (Liu et al., 2021). In addition to the demonstration of the RBBB conduction delay pattern of paced QRS, at least one of the other criteria should be demonstrated to conform to the direct capture of the LB or its branches, including recording LB potentials with LB‐local ventricular electrogram interval of 20–35 ms, demonstration of QRS morphology transition from nonselective to selective LB capture or nonselective to septal capture with incrementing output, peak activation time of LV in leads V5–V6 should be <80 ms, or programmed deep septal stimulation to demonstrate a refractory period of LB should be present (Ponnusamy et al., 2020).

### 4.4 Procedural techniques

The implantation tools are similar to His bundle pacing and the Medtronic 3830 SelecSecure lead (4.1 F) is used along with C315 His fixed curve sheath (Ponnusamy et al., 2020). Some countries have a deflectable C304 His sheath for patients with difficult anatomy. Preprocedural echocardiography should be done to assess the interventricular septal thickness to assess the presence of septal scar due to prior myocardial infarction or cardiomyopathy (Ponnusamy et al., 2020).

Unlike His bundle pacing where the target site is narrow, LBBAP has a wide area of lead placement on the left side of the septum (Ponnusamy et al., 2020). Contrary to His bundle pacing, there are no specific potentials to be targeted as the work is being done on the right side of the septum. Furthermore, the distal extent of the His bundle should be known before placing the LBBAP lead (Ponnusamy et al., 2020).

C315 His sheath to stick in the interventricular septum orientation is the success factor for LBBAP. Previous knowledge about the depth of the interventricular septum and the length of the radiopaque segment of 3830 lead is important to determine the safe depth of the lead in the septum (Ponnusamy et al., 2020).

The success rate of LBBAP is reported to be between 80.5% and 97%. The reasons for failure include inadequate sheath support, malpositioned sheath orientation, failure to lodge the lead deep into the septum, presence of septal scar, or entanglement of septal tricuspid leaflet (Li et al., 2019; Vijayaraman et al., 2019).

### 4.5 Role of ICD with LBBAP

In the studies included in this review, in the patients who had LBBB with normal PR interval, the mono‐ventricular pacing via the left bundle allowed proper atrioventricular narrowing and ventricular reverse remodeling with optimized atrioventricular delay (Clementy et al., 2022; Huybrechts et al., 2023; Ponnusamy et al., 2022). The rate of responders was 40%–50% super‐responders at 6 months in Clementy et al. (2022) at 6 months and up to 61% at 1 year.

The use of DF‐1 dual‐chamber ICD in patients with sinus rhythm, and single‐chamber ICD with atrial fibrillation had several advantages. There was a definite cost reduction because LBBAP generators are more expensive (Clementy et al., 2022; Huybrechts et al., 2023). Furthermore, there was battery longevity with simple mono‐ventricular pacing, although LV pacing‐only algorithms can also extend the battery longevity (Clementy et al., 2022). The only downside to this is that all heart failure algorithms cannot be included in this, which may be useful in this population.

**FIGURE 2:**
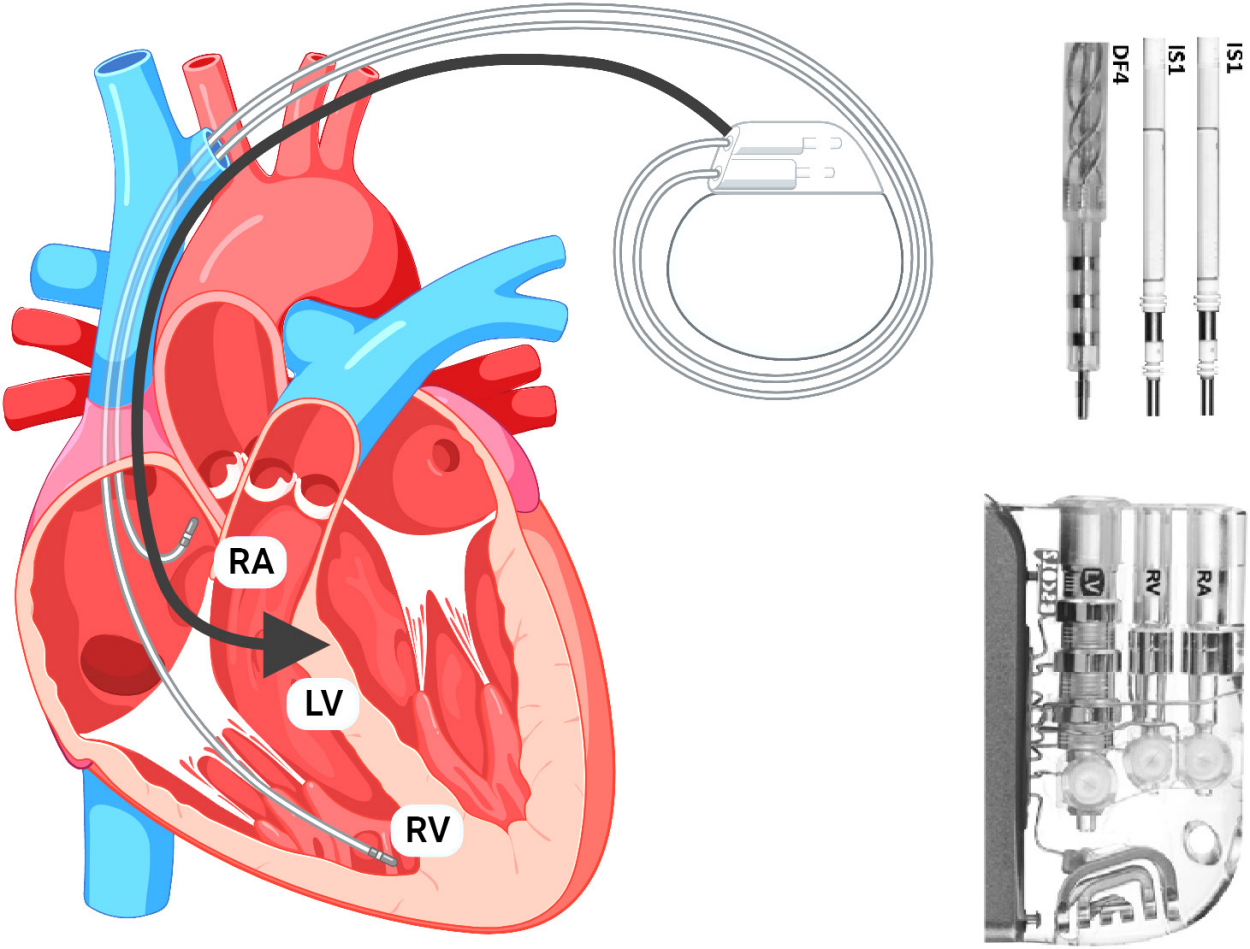
Setup of LBB‐ICD device with DF‐4 generator.

LBBAP lead, when inserted deep in the interventricular septum was not tested for ventricular fibrillation detection before these investigations. Clementy et al. show that VF detection is accurate with a bipolar lead positioned in the interventricular septum and all electrical parameters are reliable and stable over a period of time. Defibrillation is performed through an apical coil and a can, ensuring a 5 J safety margin, and is 99% reliable in the long term. Figure 2 shows the setup for the DF‐4 LBB‐ICD device.

### 4.6 Limitations

While the systematic review provides valuable insights into the use of dual‐chamber ICD for left bundle branch area pacing, there are several limitations to consider. First, the small sample size of the included studies, with only 26 patients undergoing ICD implantation for left bundle branch pacing, limits the generalizability of the findings. Additionally, the studies were conducted in only three countries, which may not reflect the outcomes of this intervention in other populations or healthcare settings. Second, all the studies were prospective cohort studies, which may be subject to bias and confounding factors. Randomized controlled trials are generally considered the gold standard for evaluating the efficacy of interventions. Third, the duration of follow‐up varied across the studies, with the shortest being 3 months and the longest being 12 months. Longer‐term follow‐up is necessary to determine the durability of the benefits of this intervention and to identify any potential complications that may arise over time. Finally, the systematic review did not include any studies that compared the use of dual‐chamber ICD for left bundle branch area pacing with other pacing strategies or interventions, such as cardiac resynchronization therapy. Therefore, it is unclear how this intervention compares to other treatment options for patients with cardiac arrhythmias.

### 4.7 Future directions

The studies included in this review were small prospective cohort studies. Larger, randomized controlled trials could provide more robust evidence for the safety and efficacy of left bundle branch area pacing with dual‐chamber ICDs. The studies included in this review only reported short‐term outcomes. Long‐term follow‐up studies are needed to evaluate the durability of left bundle branch area pacing and to determine whether it leads to improved clinical outcomes over time. Left bundle branch area pacing is one of several emerging pacing techniques. Future studies could compare the efficacy and safety of left bundle branch area pacing with other techniques, such as His‐bundle pacing and traditional right ventricular pacing.

## 5 CONCLUSION

Overall, the findings of the systematic review suggest that dual‐chamber ICD for LBBAP is a promising intervention for patients with heart conditions. The procedure offers significant improvements in QRS duration and LVEF, and there were no lead‐related complications or arrhythmic events recorded in any of the studies. However, as the review included only three studies, further research is needed to confirm the effectiveness and safety of this procedure.

## AUTHOR CONTRIBUTION

J.M and A.M contributed to the concept. S.N, H.A.R, and A.W.H contributed to the methodology. F.T, D.K, and S.M.J.Z contributed to the literature search. A.M contributed to the tables. J.M contributed to the supervision. S.N, D.K, and A.W.H contributed to the first draft. S.M.J.Z and J.M contributed to the final draft.

## CONFLICT OF INTEREST STATEMENT

The authors declare no competing interests.

## ETHICS STATEMENT

No ethical approval required as no new data was generated.

## Data Availability

Data sharing not applicable to this article as no datasets were generated or analysed during the current study.
